# Exploratory clinical predictors of prolonged hospital stay in patients with patent foramen ovale undergoing transthoracic echocardiography- or X-ray-guided closure

**DOI:** 10.1097/MD.0000000000051265

**Published:** 2026-10-30

**Authors:** Peng Zhang, Meng Chen, Xinhuan Dang, Fangfei Guo, Xiaodong Hou, Guolei Zhou, Yanling Yang, Xuan Wei, Tao You

**Affiliations:** a Department of Cardiac Surgery, Gansu Provincial People’s Hospital, Lanzhou, Gansu Province, China.

**Keywords:** Clinical prediction model, Machine learning, Patent foramen ovale, Transthoracic echocardiography, X-ray

## Abstract

Patent foramen ovale (PFO) can be managed using different image-guided treatment modalities; however, the determinants of prolonged hospitalization remain insufficiently defined. This retrospective study aimed to identify clinical predictors associated with length of hospital stay and to develop a machine learning model for predicting prolonged hospitalization in patients with PFO. Clinical data from 216 patients with PFO treated at Gansu Provincial People’s Hospital were retrospectively analyzed. Among them, 106 patients underwent transthoracic echocardiography (TTE)-guided treatment and 110 underwent X-ray-guided treatment. According to the median length of stay, patients were divided into a short-stay group (≤7 days) and a prolonged-stay group (>7 days). Baseline variables were compared between groups. Least absolute shrinkage and selection operator (LASSO) regression was used for feature selection. Five machine learning algorithms were then developed and compared in the training cohort, followed by validation in an independent validation cohort. Thirteen baseline variables showed significant between-group differences. LASSO regression retained 10 candidate predictors for model construction. Among the evaluated models, the random forest model showed the best overall performance. In the validation cohort, the model achieved an accuracy of 0.692, a precision of 0.708, a recall of 0.850, an F1-score of 0.773, and an AUC of 0.711, indicating moderate discrimination. The random forest model demonstrated moderate predictive discrimination for prolonged hospital stay. Given the limited sample size and single-center retrospective design, the model should be considered exploratory and requires external validation in larger independent cohorts before clinical use.

## 1. Introduction

Patent foramen ovale (PFO) is a common interatrial communication that persists after birth and is present in approximately 20 to 25% of adults. Although PFO is often asymptomatic, it has been associated with clinically important conditions such as cryptogenic stroke and other embolic events. In appropriately selected patients, transcatheter PFO closure is an established treatment strategy.^[1–5]^

PFO closure may be performed under different imaging-guidance modalities, including X-ray guidance and transthoracic echocardiography (TTE) guidance. X-ray guidance provides clear visualization of interventional devices but involves radiation exposure, whereas TTE guidance avoids ionizing radiation and permits real-time cardiac imaging. Both approaches are used in clinical practice, and the choice of guidance modality may depend on patient characteristics, anatomical features, procedural considerations, and institutional experience.^[6–10]^

Length of hospital stay is an important perioperative outcome because prolonged hospitalization may reflect greater clinical complexity, delayed recovery, additional monitoring or treatment requirements, and increased healthcare resource utilization. Early identification of patients at risk of prolonged hospitalization may facilitate perioperative risk stratification, discharge planning, resource allocation, and communication with patients and families. However, factors associated with prolonged hospitalization after PFO treatment remain insufficiently characterized, and individualized prediction of hospitalization duration in this population has received limited attention.^[11–17]^

Machine-learning methods may help identify complex relationships among routinely collected clinical variables and provide exploratory tools for risk prediction. Length of hospital stay is an important perioperative outcome because prolonged hospitalization may reflect greater clinical complexity, delayed postoperative recovery, additional monitoring or treatment requirements, and increased use of healthcare resources. Early identification of patients at risk of a longer hospital stay may therefore assist clinicians in perioperative risk stratification, discharge planning, allocation of hospital resources, and communication with patients and families. However, factors associated with prolonged hospitalization after treatment for patent foramen ovale have not been well characterized, and individualized prediction of hospital stay in this population remains insufficiently studied.^[18,19]^

Accordingly, the present study aimed to explore clinical factors associated with prolonged hospitalization in patients with PFO undergoing TTE-guided or X-ray-guided treatment and to evaluate whether routinely available clinical variables could provide preliminary predictive information regarding hospital stay.

## 2. Materials and methods

### 2.1. Inclusion of researchers

his retrospective single-center study consecutively included patients with patent foramen ovale (PFO) who underwent PFO closure at Gansu Provincial People’s Hospital between January 2021 and December 2024. Patients were identified from the institutional electronic medical record and procedural databases.

Patients were eligible for inclusion if they met all of the following criteria: a confirmed diagnosis of PFO; underwent PFO closure using either transthoracic echocardiography (TTE)-guided or X-ray-guided treatment at our institution during the study period; and complete clinical, perioperative, and hospitalization data were available for analysis.

Patients were excluded if they had undergone previous PFO closure or repeat intervention, underwent additional major cardiac procedures during the same hospitalization that could substantially affect the length of stay, or had incomplete key clinical or outcome data.

A total of 278 consecutive patients were assessed for eligibility between January 2021 and December 2024. After exclusion of 62 patients, 216 patients were included in the final analysis, including 106 patients who underwent TTE-guided treatment and 110 patients who underwent X-ray-guided treatment.

No missing data were present for the clinical variables included in the present analysis; therefore, no data imputation was required.

The study protocol was reviewed and approved by the Medical Ethics Committee of Gansu Provincial People’s Hospital. All study procedures were conducted in accordance with the relevant ethical standards and applicable guidelines and regulations.

The choice of TTE-guided or X-ray-guided treatment was made according to routine clinical practice and was not randomized. Treatment selection was based on the attending physician’s clinical judgment, patient-specific anatomical and clinical characteristics, procedural considerations, and other factors relevant to routine care. Therefore, differences between the 2 treatment groups may reflect underlying differences in patient characteristics and treatment selection rather than an independent effect of the guidance modality itself.

### 2.2. Defining variables

The primary outcome was prolonged hospital stay. Length of hospital stay was defined as the number of calendar days from hospital admission to discharge. In the present cohort, the median length of stay was 7 days. Because no universally accepted threshold for prolonged hospitalization has been established specifically for patients undergoing PFO closure, we used the cohort median as a prespecified pragmatic threshold for the exploratory binary analysis. Accordingly, patients were classified as having a shorter hospital stay (≤7 days) or prolonged hospital stay (>7 days).

This threshold was intended to provide an approximately balanced outcome distribution for exploratory model development rather than to represent a clinically validated cutoff. Therefore, the > 7-day definition should be interpreted as a study-specific operational definition.

Clinical information: All clinical data and subgroups are shown in Table 1.

**Table 1:** Baseline feature analysis of participants.

	Level	>7	≤7	*P*
n		82	134	
age (%)	<= 50	62 (75.6)	89 (66.4)	.202
	> 50	20 (24.4)	45 (33.6)	
gender (%)	Female	38 (46.3)	70 (52.2)	.483
	Male	44 (53.7)	64 (47.8)	
height (mean (SD))		135.26 (30.61)	152.78 (23.26)	<.001
weight (mean (SD))		40.05 (23.08)	52.80 (20.53)	<.001
BMI (mean (SD))		19.44 (4.54)	21.33 (4.23)	.002
Foramen ovale (%)	≥4mm	8 (9.8)	15 (11.2)	.217
	0–2mm	32 (39.0)	37 (27.6)	
	2–4mm	42 (51.2)	82 (61.2)	
Infarction of the brain (%)	1	3 (3.7)	3 (2.2)	.85
	2	79 (96.3)	131 (97.8)	
Demyelinating disease of the white matter (%)	1	3 (3.7)	1 (0.7)	.307
	2	79 (96.3)	133 (99.3)	
VSD (%)	1	12 (14.6)	0 (0.0)	<.001
	2	70 (85.4)	134 (100.0)	
ASD (%)	1	66 (80.5)	132 (99.2)	<.001
	2	16 (19.5)	1 (0.8)	
Patent ductus arteriosus (%)	1	4 (4.9)	1 (0.7)	.135
	2	78 (95.1)	133 (99.3)	
Foaming experimental (%)	1	0 (0.0)	1 (2.6)	.532
	2	0 (0.0)	3 (7.9)	
	3	11 (100.0)	34 (89.5)	
ECG (%)	1	67 (81.7)	88 (66.7)	.025
	2	15 (18.3)	44 (33.3)	
Coronary ARTERY STUDIES (%)	1	15 (100.0)	73 (97.3)	1
	2	0 (0.0)	2 (2.7)	
CT (%)	1	2 (50.0)	4 (66.7)	1
	2	2 (50.0)	2 (33.3)	
WBC (mean (SD))		7.10 (2.38)	5.77 (1.71)	<.001
RBC (mean (SD))		5.06 (3.67)	4.64 (0.52)	.194
Hb (mean (SD))		133.64 (21.12)	136.40 (20.69)	.347
PTL (mean (SD))		258.74 (134.49)	220.89 (74.94)	.009
INR (mean (SD))		1.04 (0.11)	1.07 (0.85)	.735
PT (mean (SD))		13.50 (1.11)	12.98 (1.55)	.009
APTT (mean (SD))		33.00 (9.34)	28.25 (12.62)	.004
FIB (mean (SD))		3.46 (3.43)	2.53 (0.63)	.002
Blood type (%)	1	21 (32.3)	24 (40.7)	.325
	2	22 (33.8)	12 (20.3)	
	3	7 (10.8)	5 (8.5)	
	4	15 (23.1)	18 (30.5)	
Duration of surgery (%)	>50	41 (50.0)	47 (35.1)	.043
	≤50	41 (50.0)	87 (64.9)	
Postoperative Antibiotics (%)	1	58 (72.5)	80 (60.2)	.093
	2	22 (27.5)	53 (39.8)	
Plavix (%)	1	19 (31.7)	37 (48.1)	.078
	2	41 (68.3)	40 (51.9)	
Treatment (%)	1	48 (58.5)	58 (43.3)	.042
	2	34 (41.5)	76 (56.7)	

### 2.3. Machine learning model building and model performance evaluation

Least Absolute Shrinkage and Selection Operator (LASSO) regression was constructed using the glmnet package (v 4.1-4)^[20]^ to select the characteristic variables. Patients were stochastically classified into 2 groups, with 70% as the training cohort and 30% as the validation cohort (both the training and validation cohorts were required to contain patients with PFO from both surgical procedures). Five common machine learning methods were applied: Extreme Gradient Boosting (XGBoost) (xgboost package) (v 1.7.3.1), RF (randomForest package) (v 4.7.1.1),^[21]^ Support Vector Machine (SVM) (e1071 package) (v 1.7-13), Logistic regression (LR) (autoReg package) (v 0.3.4), and Naive Bayes (NB) (e1071 package) (v 4.7.1.1) to develop models in the training cohort. The efficacy of the models was appraised by comparing the accuracy, precision, recall, F1 score with the caret package (v 6.0.93) and area under the curve (AUC) value (AUC > 0.7) with the pROC package (v 1.18.0),^[22]^ the best model was selected in the training cohort and validated in the validation cohort.

### 2.4. Statistical analysis

For the account of baseline characteristics, categorical variables were exhibited as counts and percentages, and distinctions between groups were analyzed by weighted chi-square tests in the tableone package (v 0.13.2). Continuous variables were denoted as mean ± standard deviation (SD) or median and interquartile spacing (IQR), and the Wilcoxon rank sum test was employed to compare the discrepancies among groups (*P *< .05). Analyses were performed utilizing the R programming language (v 4.2.2).

## 3. Results

### 3.1. Baseline statistics for PFO patients

To analyze whether there were differences in baseline characteristics (28 variables) between participants in the short hospitalization group (≤ 7 days) and the long hospitalization group (> 7 days), significant differences were found in 13 variables exemplified by height, weight, body mass index (BMI), and ventricular septal defect (VSD) (*P *< .05) (Table 2).

**Table 2:** The performance of the best model on the validation queue.

Indicator	RF
Accuracy	0.6923077
Precision	0.7083333
Recall	0.85
F1-score	0.7727273

### 3.2. Ascertainment of characteristic variables

LASSO regression analysis was performed on baseline characteristics (28 variables) based on clinical data, using length of stay as the outcome variable. When the optimal Lambda was 0.006369128, 10 baseline characteristics were finally obtained (height, cerebral infarction, cerebral white matter demyelinating disease, VSD arterial catheter failure, preoperative white blood cell, preoperative red blood cells, preoperative hemoglobin (Hb), preoperative posterior tricuspid leaflet (PTL), and treatment modality); these variables were defined as characteristic variables (Fig. 1A, B).

**Figure 1.**
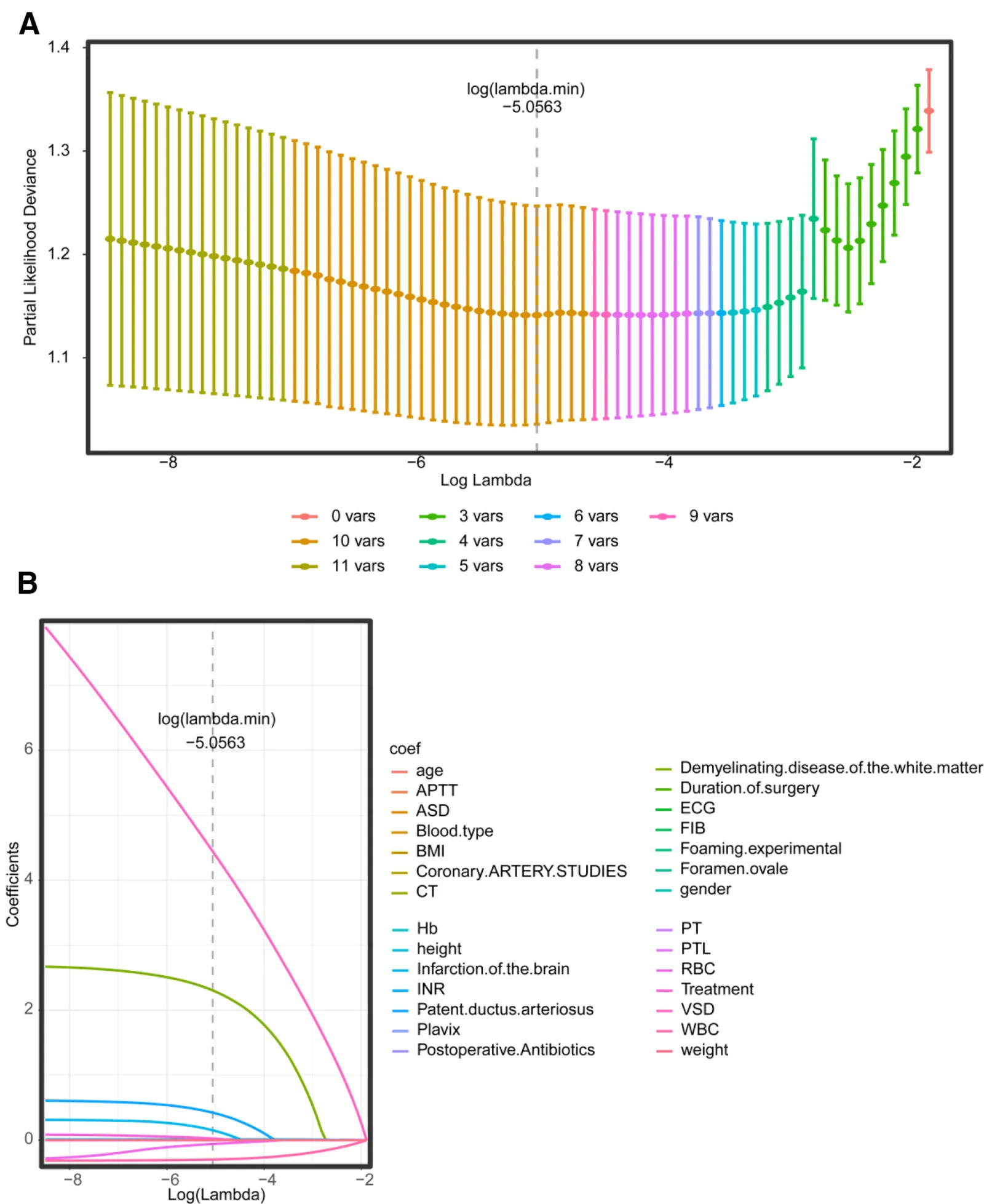
Ascertainment of characteristic variables. (A) Cross validation diagram of Lasso analysis. The optimal lambda value was at the lowest point of the red curve, where the model error was minimized. (B) Lasso coefficient spectrum. As lambda increased, the variable coefficient approached 0. When reaching the optimal lambda, variables with coefficients equal to 0 were removed.

### 3.3. Machine learning model performance comparison

To develop a machine learning model aimed at making predictions for the development of PFO patients after hospital admission, PFO patients were arbitrarily categorized into 2 groups, 70% as the training cohort and 30% as the validation cohort. In the training cohort, 5 machine learning models – XGBoost, RF, SVM, LR, and NB – based on 10 feature variables, were used to construct a prediction model for predicting the length of stay of PFO patients. ROC curves of the 5 models in the training cohort were plotted, and an AUC > 0.7 indicated that the models showed outstanding predictive efficacy. In the training cohort, there were 4 models with AUC > 0.7, namely XGBoost, SVM, LR, and RF (Fig. 2A). Subsequently, the predictive capability of the model was assessed using accuracy, precision, recall, and F1 score (the higher the level of accuracy, precision, recall, and F1 score, the superior the predictive capability). It could be seen that the RF model had the best prediction capability (Fig. 2B). To verify the predictive potency of the best RF model, accuracy, precision, recall, F1-score, and AUC values (AUC > 0.7) were used to assess the predictive capability of the RF model in the validation cohort (Fig. 2C, Table 3).

**Table 3:** Clinical data information table.

covariate	definition
age	1 ≤ 50 y, 2 > 50 y
gender	1 = male, 2 = female
height (cm)	continuous variable
weight (kg)	continuous variable
BMI	continuous variable
Foramen ovale (mm)	1 = 0–2, 2 = 2–4, 3 ≥ 4
Infarction of the brain	1 = yes, 2 = no
Demyelinating disease of the white matter	1 = yes, 2 = no
VSD	1 = yes, 2 = no
ASD	1 = yes, 2 = no
Patent ductus arteriosus	1 = yes, 2 = no
Foaming experimental	1 = GradeI, 2 = GradeII, 3 = GradeIII
ECG	1 = normal, 2 = abnormal
Coronary ARTERY STUDIES	1 = Coronary Angiography, 2 = Coronary Computed Tomographic Angiography (Coronary CTA)
CT	1 = no abnormality, 2 = with abnormality
WBC	continuous variable
RBC	continuous variable
Hb	continuous variable
PTL	continuous variable
INR	continuous variable
PT	continuous variable
APTT	continuous variable
FIB	continuous variable
Blood type	1 = A, 2 = B, 3 = AB, 4 = O, 5 = special
Treatment	1 = Treatment guided by Transthoracic Echocardiography (TTE), 2 = Treatment guided by X-ray
Duration of surgery (min)	1 ≤ 50, 2 > 50
Postoperative Antibiotics	1 = Oral medications, 2 = Intravenous injection
Plavix	1 = yes, 2 = no

**Figure 2.**
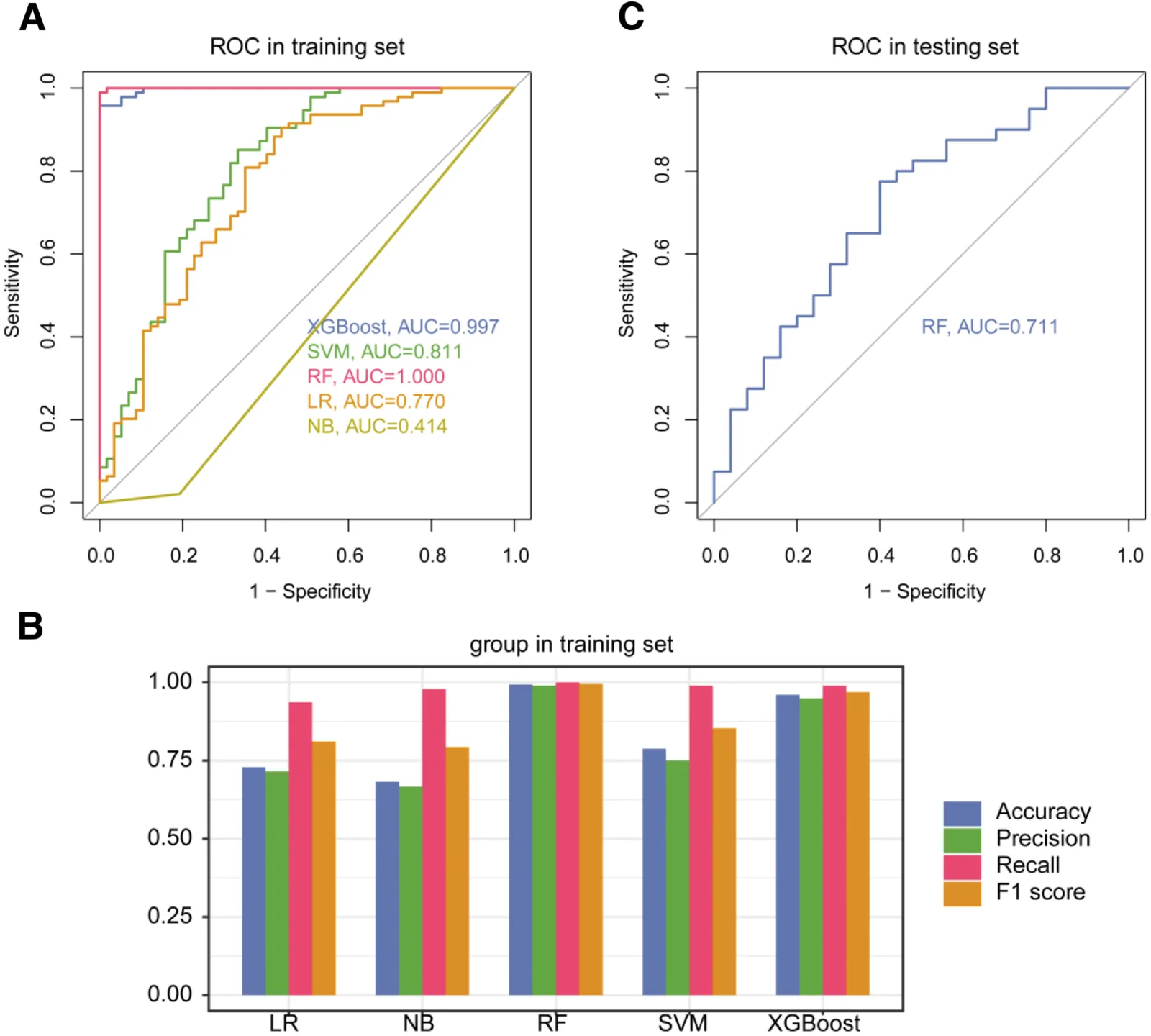
Machine Learning model performance comparison. (A) ROC curve of machine learning model in the training set. The closer the AUC was to 1, the more accurate the diagnosis was. (B) Performance of machine learning models in the training set. Blue: Accuracy; Green: Precision; Pink: Recall; Orange: F1 score. (C) ROC analysis of the best model validation queuein the validation set. The closer the AUC was to 1, the more accurate the diagnosis was.

Considering the limited sample size, model performance was evaluated using resampling-based internal validation rather than relying solely on a single random data split. Among the five candidate algorithms, the random forest model showed the most favorable overall performance. However, its predictive discrimination remained moderate rather than excellent. Therefore, the model should be interpreted as exploratory and requires validation in a larger independent cohort before clinical application.

### 3.4. Importance ranking of characteristic variables

Further information on the importance of 10 characteristic variables for the best RF model showed that height, VSD, and preoperative WBC were the top 3 important characteristics (Fig. 3).

**Figure 3.**
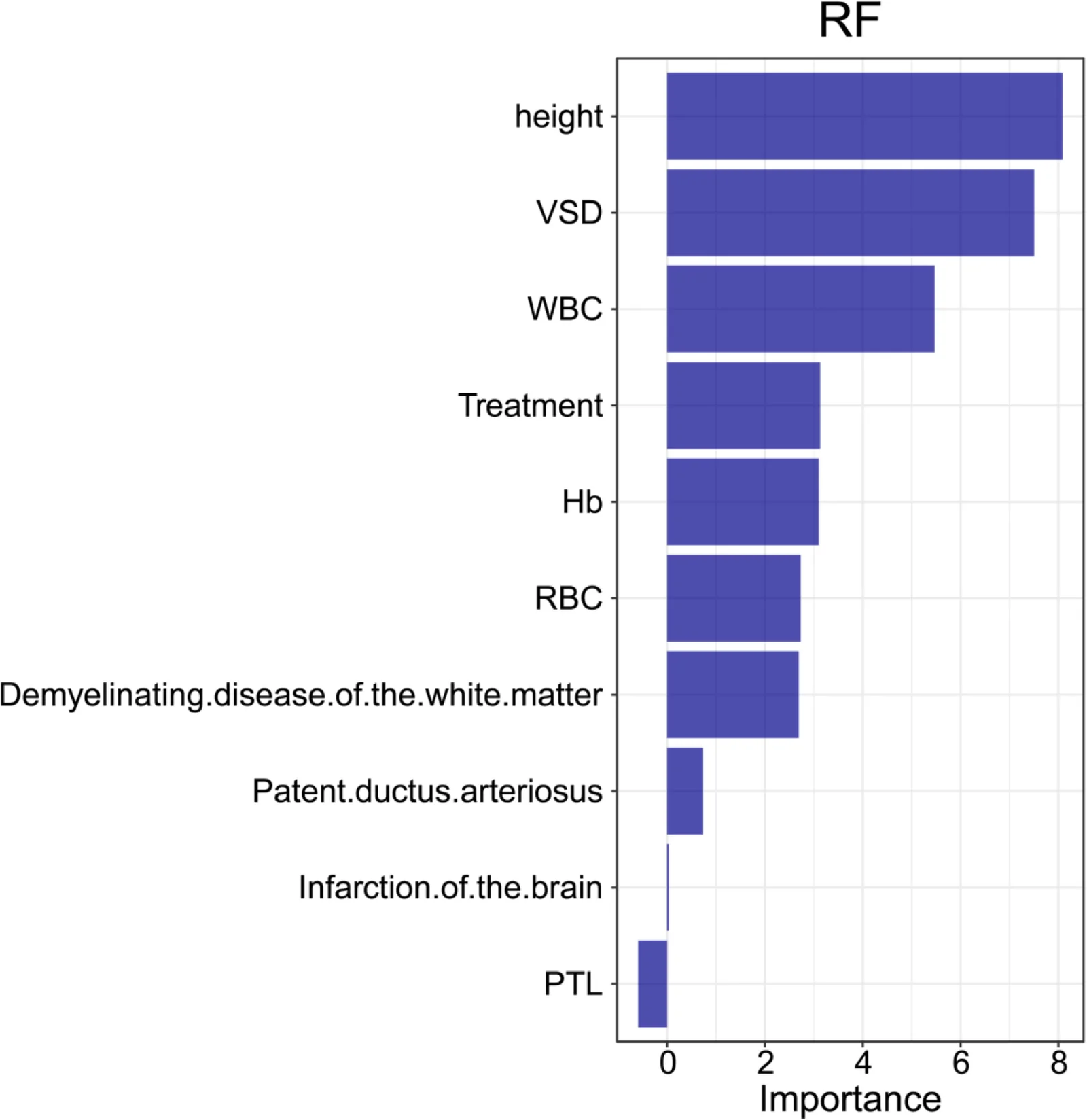
Feature variable selection for the best model. The importance of feature variables is ranked from top to bottom.

## 4. Discussion

The foramen ovale is the normal pathway between the right and left atrial septa during fetal life in humans.^[23,24]^ Before birth, the foramen ovale is kept open because blood flow is from right to left. After birth, normal pulmonary circulation is established, and the foramen ovale closes as the pressure within the atria increases, forcing the lamina of the primary atrial septum to press against the surface of the foramen ovale.^[25,26]^ Which is associated with congenital heart disease.^[27]^In the management of PFO, transthoracic echocardiography (TTE) and X-rays are required to guide the closure procedure.^[28,29]^ In this study, a clinical characterization model was constructed to predict the length of hospital stay in patients with PFO based on clinical data from patients with PFO who underwent TTE and X-ray-guided treatment.

In this study, the length of stay of PFO patients was divided into short and long groups based on the median length of stay (7 days). Thirteen baseline characteristics of PFO patients differed significantly between the 2 treatment modalities, TTE and X-ray,^[30]^ may be more accurate in locating surgical instruments in some complex cases^[31]^; TTE-guided treatment is relatively safe, but may be more effective in some cases. TTE-guided therapy is relatively safe, but may be limited by the patient’s physical condition in some cases.^[32–35]^

VSD is one of the most common congenital heart diseases (CHD) and accounts for up to 40% of all congenital heart malformations. VSD can occur either as a stand-alone congenital heart disease or in association with other congenital heart malformations. Chromosomal abnormalities (e.g., aneuploidy and structural variants) as well as rare point mutations in various genes have been reported to be associated with this heart defect.^[36]^ Cases of VSD combined with PFO are not uncommon, and surgical or minimally invasive occlusion can be used to address the above congenital heart defects effectively, with benefit to the patient.^[37–39]^ A possible association between PFO failure and VSD has been reported, and this association may be because both ventricular septum and PFO development take place during embryonic cardiac development and are influenced by the same genetic and environmental factors.^[40]^

PFO is a well-known risk factor for stroke, while on the other hand, it seems to be associated with migraine with aura.^[41,42]^ In recent years, numerous correlations between migraine, ischemic stroke, and PFO have been reported.^[43–46]^PFO, In this study, cerebral infarction was identified as a characteristic variable of PFO by Lasso regression analysis, which implies an association between cerebral infarction and PFO, and closure of the foramen ovale has the potential to benefit such patients.^[40,47–50]^

In this study, patients with PFO were categorized into short and long hospitalization groups based on the median length of hospitalization, and 13 baseline characteristics of patients with PFO were found to be significantly different among different treatments. Based on the clinical data of PFO patients who underwent transthoracic echocardiography and X-ray guided therapy, we investigated whether different treatments were associated with the length of hospitalization in PFO patients. We also constructed an interpretable optimal model for predicting the length of hospitalization in patients with PFO using machine learning and compared the influence of the 2 treatments on predicting the length of hospital stay in patients with PFO. In this study, five machine learning models (XGBoost, RF, SVM, LR, and NB) were used to construct a model for predicting the length of hospitalization of patients with PFO.ROC curves were plotted using the R package “pROC” to assess the predictive performance of the models, Although the random forest model showed the highest apparent performance among the candidate algorithms, its near-perfect performance in the development data suggests potential overfitting. In contrast, the internally validated discrimination was only moderate. Therefore, the observed performance should be interpreted cautiously, and the model should not be considered ready for clinical implementation.. The RF model has long been applied in studies related to PFO, and Laura Oliva used the RF model to differentiate between PFO and ASD patient populations in healthcare management data.^[51,52]^ In this study, we first used the RF model to predict the length of hospitalization for patients and achieved good results, providing an opportunity for clinical healthcare resources to improve the quality of hospitalization. This study is the first to use an RF model to predict the length of hospital stay of patients, and achieved good results, providing a valuable reference for the optimal allocation of clinical medical resources.

Several limitations should be acknowledged. First, the sample size was limited to 216 patients from a single center, which restricts the statistical stability and generalizability of the machine-learning models. In the original analysis, further division of the dataset into training and validation subsets reduced the effective number of observations available for model development and may have increased the risk of overfitting and unstable performance estimates. Therefore, the present model should be regarded as exploratory and hypothesis-generating rather than as a definitive clinical prediction tool. Although resampling-based internal validation can improve the efficiency of model assessment in small datasets, it cannot replace external validation. Future studies should validate the model in substantially larger, independent, multicenter cohorts before clinical implementation. The definition of prolonged hospitalization also warrants consideration. We dichotomized length of stay at 7 days because this represented the median value in the present cohort and provided a pragmatic threshold for exploratory classification. However, this cutoff is study-specific and has not been established as a clinically validated threshold for patients undergoing PFO closure. Dichotomization of a continuous outcome may also result in loss of information and may reduce statistical efficiency. Therefore, future studies with larger datasets should consider modeling length of stay as a continuous or time-to-discharge outcome and should evaluate clinically meaningful thresholds in independent populations.

## 5. Conclusion

The random forest model demonstrated acceptable performance for predicting prolonged hospital stay in patients with PFO, with moderate discrimination in the validation cohort. Given the limited sample size and retrospective single-center design, these findings should be considered exploratory and require external validation before clinical implementation.

## Author contributions

**Conceptualization:** Peng Zhang, Meng Chen, Xinhuan Dang, Fangfei Guo, Xiaodong Hou, Guolei Zhou, Yanling Yang, Xuan Wei, Tao You.

**Data curation:** Peng Zhang, Meng Chen, Xinhuan Dang, Fangfei Guo, Xiaodong Hou, Guolei Zhou, Yanling Yang, Xuan Wei, Tao You.

**Formal analysis:** Peng Zhang, Meng Chen, Xinhuan Dang, Fangfei Guo, Xiaodong Hou, Guolei Zhou, Yanling Yang, Xuan Wei, Tao You.

**Funding acquisition:** Tao You.

**Investigation:** Tao You.

**Writing – original draft:** Tao You.

**Writing – review & editing:** Tao You.
